# Ultralow energy photoacoustic microscopy for ocular imaging *in vivo*

**DOI:** 10.1117/1.JBO.25.6.066003

**Published:** 2020-06-09

**Authors:** Wei Zhang, Yanxiu Li, Van Phuc Nguyen, Katherine Derouin, Xiaobo Xia, Yannis M. Paulus, Xueding Wang

**Affiliations:** a University of Michigan, Department of Biomedical Engineering, Ann Arbor, Michigan, United States; b University of Michigan, Department of Ophthalmology and Visual Sciences, Ann Arbor, Michigan, United States; c Central South University, Eye Center of Xiangya Hospital, Changsha, China; d Central South University, Hunan Key Laboratory of Ophthalmology, Changsha, China

**Keywords:** photoacoustic microscopy, ocular imaging, laser safety, high sensitivity

## Abstract

**Significance:** The development of ultralow energy photoacoustic microscopy (PAM) on the clinically relevant pigmented rabbit eye model paves a road toward translation of the emerging PAM technology in ophthalmology clinics.

**Aim:** Since the eye is particularly vulnerable to laser damage, we aim to develop an ultralow energy PAM system to significantly improve the laser safety of PAM by increasing the sensitivity of the system and reducing the incident laser energy for imaging.

**Approach:** A multichannel data acquisition circuit with two-stage signal amplification was specially designed, which, in combination with the application of 3 by 3 median filter in the spatial domain, significantly improved the signal-to-noise ratio of the PAM system. The safety of this system was validated by histopathology, fluorescein angiography, and fundus photography.

**Results:** Experiments performed on pigmented rabbits demonstrated that, when using this ultralow energy PAM system, satisfactory image quality can be achieved in the eye with an incident laser fluence that is only 1% of the American National Standards Institute safety limit. Fundus photography, fluorescein angiography, and histopathology were performed after the imaging procedure, and no retinal or ocular damage was observed.

**Conclusions:** The proposed ultralow energy PAM system has excellent safety and holds potential to be developed into a clinical tool for ocular imaging.

## 1 Introduction

Due to the optical transparency of the eye, optical imaging methods are highly beneficial in the field of ophthalmology for diagnosis. Current clinically available optical imaging modalities include fundus photography, fluorescein angiography (FA),^1^ indocyanine green angiography,^2^ optical coherence tomography (OCT),^3^ OCT angiography (OCT-A),^4^ and scanning laser ophthalmoscopy.^5^ As a novel biomedical imaging method, photoacoustic microscopy (PAM) has the unique capability to noninvasively explore the optical absorption properties in biological tissues with high spatial resolution and deep penetration.^6^ In PAM, a nanosecond-pulse-duration laser beam is used to induce localized thermoelastic tissue expansion. The thermoelastic wave emitted from the target area can be detected by an ultrasonic transducer(s) to extract the optical absorption information of the targeted area.^7^ Previous publications including those from our group have described the basic concept of a PAM ocular imaging system and investigated its potential applications and unique advantages in ophthalmic imaging.^8^^–^^11^

**Fig. 1:**
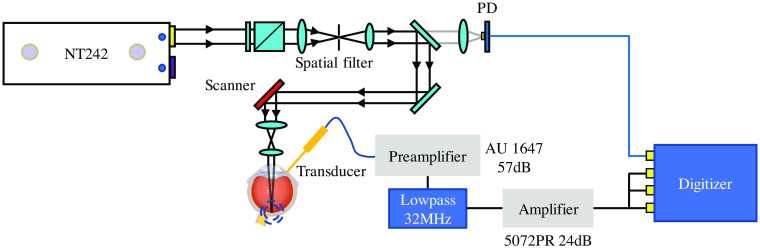
Ultralow energy PAM imaging and DAQ system. PD, photodiode.

Laser safety in the visible and near-infrared spectral bands is an incredibly important aspect in ocular imaging. The transparent eye allows laser light transmission to the posterior segment, which also means that most of the laser energy will be directly delivered to the photoreceptors.^12^ Another reason for the sensitivity of the retina to light damage is due to the focusing of incoming light rays on the retina by the eye’s optical system. Since the photoreceptors, which are the neurons at the posterior portion of the retina, are extremely sensitive to light, the eye is particularly vulnerable to laser damage.^13^ Although previous studies have suggested that PAM imaging of the eye can be achieved using laser fluence lower than the safety limits from the American National Standards Institute (ANSI),^9^ laser safety remains a concern for potential clinical translation of this technology. To further improve the safety of this technology, we have recently developed an ultralow energy PAM ocular imaging system, as shown in Fig. 1. After drastically enhancing its signal-to-noise ratio (SNR), the performance of this system working with significantly reduced laser fluence was examined via the experiments on a clinically relevant rabbit eye model *in vivo*. The safety of this system was validated by histopathology, FA, and fundus photography, paving the road toward clinical application.

## 2 Methods

### 2.1 System Design

The details regarding the optical design of our PAM ophthalmic imaging system have been described in our previous publications.^8^^,^^14^ A spatial filter was placed after a tunable attenuator to achieve an approximate Gaussian beam with a diameter of 5 mm. The pulse to pulse laser energy fluctuation was monitored and recorded by a photodiode (PD) through a beam splitter. A telescope configuration right after the two-axis scanning system was applied to achieve a parallel beam with a diameter of 1 mm before the cornea, which led to a relatively small laser spot on the retina and minimized the variation in spot size caused by the change in distance between the objective lens and the eye. A laser wavelength of 578 nm where hemoglobin has a strong optical absorption was selected for imaging. Before imaging, the incident laser energy before the cornea was measured by a standard PD power sensor (S121C, Thorlabs). Since the diameter of laser beam is smaller than the dilated pupil of rabbit eye, the measured incident laser energy before the cornea represents the total intraocular energy used for the safety calculation.

The generated photoacoustic signal was detected by a custom-built needle ultrasound transducer with a central frequency of 25.0 MHz and an aperture size of $0.7\times 0.7\;{\mathrm{mm}}^{2}$ (Optosonic Inc., Arcadia, California). The detected signal was first amplified by a 57-dB low-noise amplifier (AU-1647, L3 Narda-MITEQ, New York) and went through a low-pass filter (32 MHz, BLP-30+, Mini Circuits). The signal was then sent to a pulser/receiver (5072PR, Olympus) with programmable gain as the second stage amplifier, whose low pass filter and high pass filter were set to full BW and 1 MHz, respectively. The further amplified signal was sent to three different channels of a multichannel data acquisition (DAQ) system (PX1500–4, Signatec Inc., Newport Beach, California) with 8-bit resolution and a sampling rate of 500 MHz. To fully utilize the dynamic range of the DAQ system, the gain of the second stage amplifier was set to 24 dB, which also ensured that the maximal system noise would not go beyond 60% of the dynamic range of DAQ system. At the same time, the pulse-to-pulse laser energy monitored by the PD was digitized using the same DAQ card at the same sampling rate. We have previously reported the lateral and axial resolution of our PAM system which, by imaging test gratings, were quantified as 4.1 and 37  μm, respectively.^11^

### 2.2 Data Processing

The three signals acquired by the three channels of the multichannel DAQ system were averaged. This step can enhance the SNR by a factor of $\sqrt{3}$ because the DAQ system noises associated with the three channels are independent. After this average, the signal was then normalized by the recorded laser energy to eliminate the variation due to the laser pulse energy fluctuation. To further enhance the SNR, a 3×3 median filter in the spatial domain was applied to the signals acquired over the three-dimensional space. This step, although it may slightly reduce the spatial resolution of the imaging system, could further enhance the SNR by removing the high-frequency noise. After these data processing steps, a PAM image was then assembled from the signals acquired via the point-by-point raster scan.

### 2.3 ANSI Safety Limit

The ANSI Z136.1 laser safety standard takes into account laser wavelength, exposure duration, repetition rate, illumination spot size, and pupil diameter for ocular exposure. The limits of the maximum permissible exposure (MPE) for the three types of illuminations include single-pulse maximum permissible exposure (${\mathrm{MPE}}_{\mathrm{sp}}$), average power MPE for thermal and photochemical hazard (${\mathrm{MPE}}_{\text{average}}$), and multiple-pulse MPE for thermal hazards (${\mathrm{MPE}}_{\mathrm{mp}}$).^9^^,^^15^ The ${\mathrm{MPE}}_{\mathrm{sp}}$ for single laser pulse energy is the most conservative among the three.

The retinal MPE value depends on the angular subtense of the apparent source α. In laser scanning ocular imaging, the angular subtense of the parallel beam is determined by the air-equivalent focal length of the eye and corresponding laser spot size on the retina, which should be around 17 mm and 20 to 25  μm, respectively.^16^^,^^17^ $\alpha =\frac{25\;\mu m}{17\;\mathrm{mm}}<\alpha_{\min}$ is achieved with intrabeam exposure of the eye by such a parallel Gaussian beam, where $\alpha_{\min}=1.5\;\mathrm{mrad}$ is defined by ANSI standard for safe use of lasers in ocular imaging.^15^ The maximum permissible single laser pulse energy, ${\mathrm{MPE}}_{\mathrm{sp}}$, from a parallel Gaussian beam, as determined by the human pupil diameter of 7 mm, is 162 nJ.^11^

### 2.4 Animal Handling

All the experimental procedures were performed in accordance with the ARVO (The Association for Research in Vision and Ophthalmology) Statement for the Use of Animals in Ophthalmic and Vision Research and were approved by the Institutional Animal Care & Use Committee (IACUC) of the University of Michigan (Protocol PRO00008566, Photoacoustic & Molecular Imaging of the Eye). Five Dutch-belted rabbits (both genders, 3 to 4 months, 1.5–2.5 kg) were involved in this study. The details regarding our animal preparation can be found in our previous publication.^8^ In brief, the rabbits were first anesthetized with a mixed solution of ketamine (40  mg/kg) and xylazine (5  mg/kg) by intramuscular (IM) injection. The anesthesia was maintained by vaporized isoflurane anesthetic with 1.5% to 2% isoflurane. The pupils of the eyes were dilated before performing the PAM imaging with 2.5% phenylephrine hydrochloride and 1% tropicamide ophthalmic solutions. Topical anesthesia was applied with 0.5% topical tetracaine drops prior to initiation of the experiments. The anesthesia level and rabbit state were monitored during the imaging procedure.

After completion of all PAM imaging procedures, the retina of each rabbit eye was checked using fundus photography and FA to look for possible damage caused by the imaging procedure. Then, the rabbit was euthanized by intravenous injection of pentobarbital (Beuthanasia solution, 0.22  ml/kg I.V, 50  mg/mL) (Intervet Inc., Madison, New Jersey). The eyeballs were removed and fixed in Davidson’s fixative solution (VWR, Radnor, Pennsylvania) for 24 to 48 h. The fixed tissues were cross-sectionally cut in 5-mm sections and embedded in paraffin. Subsequently, the paraffin-embedded tissues were sliced to a thickness of 5 to 6  μm and stained with hematoxylin and eosin (H&E) for standard histopathologic evaluation.

## 3 Results

### 3.1 Imaging Experiments

**Fig. 2:**
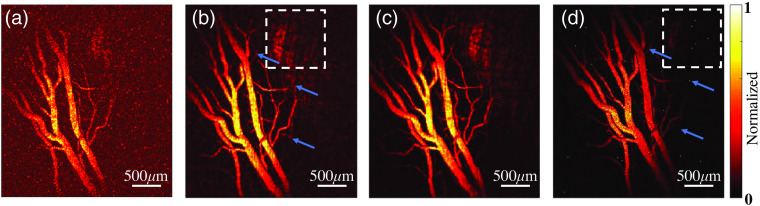
PAM images of retinal microvessels in a pigmented rabbit eye *in vivo*. (a)–(c) The images acquired by the ultralow PAM system when using 1.6 nJ (1% of ANSI safety limit), 3.2 nJ (2% of ANSI safety limit), and 4.8 nJ (3% of ANSI safety limit) of pulse energy, respectively. (d) The image acquired by our original PAM system when using 20 nJ (13% of ANSI safety limit) of pulse energy. The white dashed boxes indicate the areas for comparison; the blue arrays indicate the corresponding microvessels.

The performance of the ultralow energy PAM system was tested by imaging the retinal blood vessels in the eyes of pigmented rabbits *in vivo*. Three different pulse energy levels, including 1.6, 3.2, and 4.8 nJ, which are at 1%, 2%, and 3% of the ANSI safety limit, respectively, were used in imaging. As shown in Figs. 2(a)–2(c), at all three energy levels, the PAM system can image the retinal blood vessels with sufficient contrast-to-noise ratios. Even in the image acquired using 1.6 nJ energy (1% of the ANSI safety limit), microvessels in the retina can be recognized. The image quality was further improved when using higher pulse energy (3.2 and 4.8 nJ), as demonstrated by additional vessels presented and the higher contrast-to-background ratios achieved. However, the differences in image quality using 3.2- and 4.8-nJ laser energy are very small, suggesting that, for the current application, there is no need to use laser pulse energy beyond 2% of the ANSI safety limit.

To validate the improvement in performance, the same area in the rabbit retina was also imaged using our original PAM setup working with a laser pulse energy level of 20 nJ, as shown in Fig. 2(d). As reported in our previous publication,^8^ 20 nJ pulse energy, which is equivalent to 13% of the ANSI safety limit, was the lowest that could achieve acceptable image quality when using our original PAM setup. Compared to the image in Fig. 2(d), more microvessels (indicated by blue arrows) can be recognized in the image in Fig. 2(b). In addition, as shown in white dashed box region, more details of the retinal pigment epithelium layer can be detected with our ultralow energy PAM system. These improvements demonstrate that the ultralow energy PAM system working with 3.2 nJ of pulse energy can achieve better imaging of retinal vessels than the original PAM system working with 20 nJ of pulse energy.

**Fig. 3:**
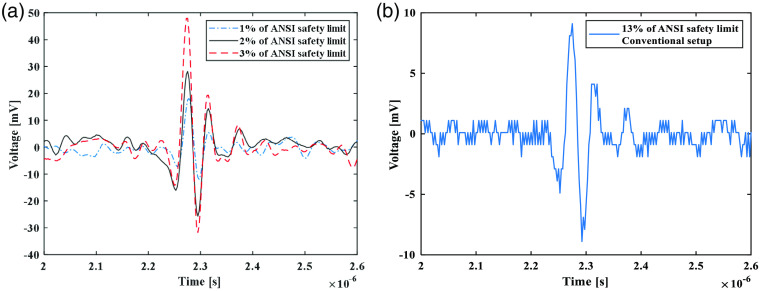
A-scan signals from the same location in the rabbit retina scanned by different setups when using different levels of laser pulse energy. (a) A-scan signals acquired by the ultra-low energy PAM system when using 1.6 nJ (1% of ANSI safety limit), 3.2 nJ (2% of ANSI safety limit), and 4.8 nJ (3% of ANSI safety limit) of pulse energy, respectively. (b) A-scan signal acquired by our original PAM setup when using 20 nJ (13% of ANSI safety limit) of pulse energy.

To further quantify the improvement in performance brought by the new design, A-scan signals from the same location were extracted from volumetric scans leading to the imaging results in Fig. 2, and then the SNR was quantified from each of the extracted A-scan signal. Figure 3(a) shows the A-scan signals from the same location scanned by the ultralow energy PAM system when using 1.6 nJ (1% of ANSI safety limit), 3.2 nJ (2% of ANSI safety limit), and 4.8 nJ (3% of ANSI safety limit) of pulse energy, respectively. The quantified SNR are 3.2, 5.8, and 8.6 dB, respectively. Figure 3(b) shows the A-scan signal from the same location scanned by our original PAM system when using 20 nJ (13% of ANSI safety limit) of pulse energy. The quantified SNR is 4.5 dB. As the SNR of PAM is proportional to the applied pulse energy, the estimated improvement in sensitivity brought by the new design is 9.2 folds.

### 3.2 Safety Evaluation

**Fig. 4:**
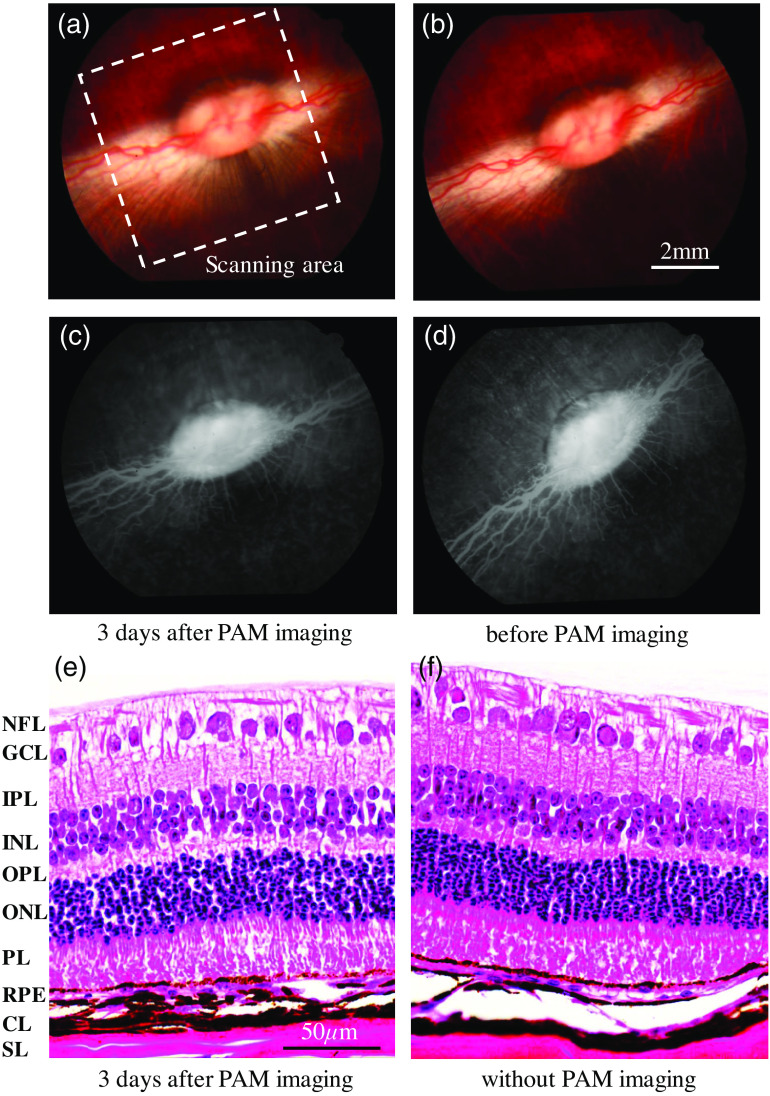
Results from safety evaluation using fundus photography, FA, and H&E-stained histopathology. (a) Fundus photograph of the retina of a pigmented rabbit eye acquired 3 days after PAM imaging. (b) Fundus photograph of the retina of a pigmented rabbit eye before performing PAM imaging (control). (c) FA image of the retina of a pigmented rabbit eye acquired 3 days after PAM imaging. (d) FA image of the retina of a pigmented rabbit eye before performing PAM imaging (control). (e) H&E histology photograph of the retina of the pigmented rabbit eye scanned by PAM. (f) H&E histology photograph of the retina of the pigmented rabbit eye that was not imaged by PAM (control). The white dashed box marks the retina area that was scanned by PAM. NFL, nerve fiber layer; GCL, ganglion cell layer; IPL, inner plexiform layer; INL, inner nuclear layer; OPL, outer plexiform layer; ONL, outer nuclear layer; PL, photoreceptor layer; RPE, retinal pigment epithelium; CL, choroidal layer; SL, scleral layer.

Fundus photography, FA, and histology were performed to evaluate possible laser damage in the pigmented rabbit eyes after performing the PAM imaging. The fundus photograph in Fig. 4(a), the FA image in Fig. 4(c), and the histology result in Fig. 4(e) were acquired 3 days after the rabbit receiving PAM imaging. In this safety evaluation, the laser pulse energy used in PAM imaging was 3.2 nJ (2% of ANSI safety limit). The retinal area scanned by PAM had a size of 7 mm by 7 mm, as marked by the white dashed box in Figs. 4(a) and 4(c). This area was also the one that was sectioned for histology examination. To be used as a control, the eye before performing PAM imaging was examined by the same procedure of fundus photography and FA, and the results are shown in Figs. 4(b) and 4(d). The eye of another pigmented rabbit without being scanned by PAM was also sectioned for histology examination, as shown in Fig. 4(f). Compared to the results from the control, the safety evaluation results from the rabbit eye acquired 3 days after PAM imaging do not show any detectable difference. Neither on the fundus photograph nor on the FA can we see any noticeable damage in the tissues before and after PAM imaging. For the H&E histology photograph, each layer demonstrates normal morphologic characteristics both with and without PAM imaging.

## 4 Discussion and conclusion

This paper presents an ultralow energy PAM system developed for ophthalmic imaging. To the best of our knowledge, it is the first time that retinal imaging can be achieved by PAM with a very low laser pulse energy of only 1% of the ANSI safety limit. By applying the two-stage signal amplification and multichannel DAQ, the dynamic range of the DAQ system was fully utilized, which helped to distinguish much more detail in the detected signal. In addition, by applying a 3 by 3 spatial-domain-based median filter, the acquired signals were averaged at each time point to further reduce the system noise. By combining the spatial average in the data processing procedure with the electrical average in the DAQ system, each A-scan received an equivalent average of 27 times. This average, unlike the time-domain signal average utilized in many previous studies to enhance the sensitivity of PAM, is not performed over multiple laser pulses and, therefore, does not sacrifice the imaging speed or raise safety concerns of multiple pulse exposure. In addition, the limited field of view of the needle detector causes decreased sensitivity to the PA response toward the periphery of the image. In the future, a ring-shaped array, which can be integrated with a contact lens, could overcome this limitation in field of view and further improve the sensitivity of PAM ocular imaging.

The experiments conducted on pigmented rabbit eyes *in vivo* demonstrated that the newly designed system and the data processing method can significantly reduce the laser pulse energy required for imaging retinal vasculature. Although the image acquired with the pulse energy at 2% of the ANSI safety limit shows better results, most of the retinal blood vessel can be clearly distinguished when using the pulse energy at 1% of the ANSI safety limit. Compared with our original PAM system developed and used in our previous studies,^8^^,^^11^ the pulse energy required for ocular imaging was reduced by 9.2 times. The excellent safety of the ultralow energy PAM system for retinal imaging was validated by fundus photography, FA, and H&E-stained histopathology conducted on the rabbit eyes at 3 days after PAM imaging. The results from these tests confirmed that the PAM imaging working with laser pulse energy at 2% of ANSI safety limit did not induce any noticeable damage in the pigmented rabbit eye.

In summary, an ultralow energy PAM system was described in this work. Using this system, PAM imaging of retinal microvessels *in vivo* in pigmented rabbit eyes was achieved using very low laser pulse energy which was 1% of the ANSI safety limit. The excellent safety of this PAM ocular imaging system was validated by fundus photography, FA, and H&E-stained histopathology. This successful study on the clinically relevant pigmented rabbit eye model paves a road toward translation of the emerging PAM technology to ophthalmology clinics.
